# The development of literacy self-concept in preschool children within the home literacy environment

**DOI:** 10.1007/s10212-026-01184-0

**Published:** 2026-09-05

**Authors:** Öykü Camligüney, Tina Schiele, Maria Valcárcel Jiménez, Frank Niklas

**Affiliations:** Department of Education and Educational Psychology, Ludwig-Maximilians-Universität München, Leopoldstraße 13, 80802 Munich, Germany

**Keywords:** Self-concept, Early literacy skills, Home literacy environment, Early childhood, Learning4Kids

## Abstract

Literacy self-concept is associated with various educational outcomes, particularly literacy skills. A positive literacy self-concept enhances literacy skills and increased skills positively influence self-concept. At preschool age, a stimulating home literacy environment may additionally contribute to a stronger relation between literacy self-concept and early literacy skills, i.e., an accurate literacy self-concept, as it offers children opportunities to apply and observe their skills. To test this idea, the current study examined the correlations between literacy self-concept and early literacy skills and the role of the home literacy environment in this relationship with moderated regressions. Data from 500 preschool children and their families over two years with four timepoints (t1-t4) were analyzed. Early literacy skills at t1 were negatively correlated with subsequent measures of literacy self-concept, but early literacy skills at t4 correlated positively with literacy self-concept. The home literacy environment was significantly associated with early literacy skills, but not with literacy self-concept. The home literacy environment was a significant moderator in the relation between early literacy skills and literacy self-concept at t2, t3, and t4. Here, stronger correlations between early literacy skills and literacy self-concept were found for children with a higher-quality home literacy environment than those with a lower-quality Home Literacy Environment. The findings offer important insights into how a stimulating home literacy environment may help children develop a positive and accurate self-concept.

## Introduction

Academic self-concept plays a pivotal role in children’s academic, social, and emotional development (Dapp & Roebers, 2018). A positive self-concept is widely regarded as a key educational goal due to its beneficial impact on academic outcomes such as motivation and performance (Marsh et al., 2005). However, an unrealistic positive self-concept can lead to misguided decisions and heightened anxiety when facing academic challenges (Carroll et al., 2009; Dunning et al., 2004). Therefore, it is important to understand how children can develop realistic and accurate academic self-concepts, i.e., self-concepts that closely align with their actual skills, but remain positive.

Self-concept is crucial for achievement within specific domains such as literacy. Here, literacy self-concept (LSC) is a strong predictor of reading and writing skills (Chapman et al., 2000; Retelsdorf et al., 2014), which are essential for knowledge acquisition, skill development, and social integration from an early age (Artelt et al., 2001). Children with a positive LSC are often motivated and engaged in related tasks, resulting in better academic skills and achievement levels (Retelsdorf et al., 2014). Additionally, children with greater literacy skills tend to have a more positive LSC based on prior experiences and feedback (Segerer et al., 2021).

Despite the well-established link between LSC and literacy skills, there is limited research on the relation between LSC and the preschool precursors of later reading and writing skills, known as early literacy skills (ELS). Additionally, not much is known about factors that may influence this relation, such as the home literacy environment (HLE). Children exposed to enriched literacy experiences within the HLE, with more opportunities to observe and practice literacy skills (Sénéchal & LeFevre, 2014), may develop a more accurate LSC than those with limited exposure. Surprisingly, the role of the HLE in the relation between ELS and LSC has been neglected so far. Addressing this gap, the current study aims to investigate the relation between LSC and literacy skills in preschool children and the role of the HLE in this relation.

### Self-concept in preschool children and early literacy skills

In general, self-concept describes beliefs or perceptions about one’s own skills, traits, and characteristics specific to a domain such as academic or social-emotional contexts (Shavelson et al., 1976). In academic contexts, individuals’ understanding and views of themselves are referred to as academic self-concept (Bong & Skaalvik, 2003). Academic self-concept includes individuals’ perceptions in different specific domains, such as literacy or mathematics. Unlike self-esteem, it is domain-specific, and unlike self-efficacy, it is not tied to specific situations or tasks (Bong & Skaalvik, 2003). It develops through interactions with the environment and is particularly shaped by feedback from significant others (Shavelson et al., 1976).

Academic self-concept is regarded as a crucial determinant of various educational outcomes, including performance (e.g., Marsh & Martin, 2011), achievement goals (e.g., Spinath & Stiensmeier-Pelster, 2003), academic interests and choices (e.g., Van Der Aar et al., 2019), effort and persistence (e.g., Marsh et al., 2016), and level of educational attainment (e.g., Marsh & O’Mara, 2008). It is also critical for a smooth transition from a play-based environment in preschool to formal education in elementary school (Dapp & Roebers, 2018). However, although there are considerable advancements in self-concept research among older children, a surprisingly limited number of investigations have been undertaken with preschool children (Arens et al., 2016; Cohrssen et al., 2016; Marsh et al., 2002).

Young children often lack the cognitive ability to effectively monitor and remember comprehensive information about themselves, including academic-related details. Consequently, they struggle to accurately predict their future academic performance because they rely on incomplete or unreliable information about their skills (Xia et al., 2022). Moreover, they often have difficulties distinguishing between their desires and actual capabilities. Therefore, they may base their performance predictions on desired outcomes rather than realistic assessments of their skills (Xia et al., 2022). As a result, young children often overestimate their academic skills, as demonstrated by various studies (Cohrssen et al., 2016; Marsh, 1990; Niklas & Schneider, 2012; Xia et al., 2022).

Early overestimation of abilities in young children has long raised questions about their ability to develop an accurate and differentiated self‑concept (Marsh, 1990; Shavelson et al., 1976). Earlier developmental models suggested that children’s self-concepts are not yet accurate in preschool and only begin to refine in later years (Shavelson et al., 1976). However, a growing body of research indicates that self-concept begins to form early in childhood and, once established, remains relatively stable over time (Arens et al., 2016; Dapp & Roebers, 2018).

Regarding the differentiation of academic self-concept across domains, i.e., mathematics and LSC, research delivers inconsistent findings for preschool children. While Ehm et al. (2013) found that the differentiation between academic domains begins from the 3rd grade on, other studies point to a differentiated self-concept even in preschool (Arens et al., 2016; Cohrssen et al., 2016; Dapp & Roebers, 2018; Verschueren et al., 2012).

In the context of literacy, preschool LSC refers to how children perceive their abilities in literacy-related subjects such as reading and writing, mastering the alphabet, and recognizing letters (Harter & Pike, 1984). The development of LSC in preschool children may depend on various factors, although research in this area is still scarce. For example, some evidence suggests that girls tend to have a higher LSC than boys (Marsh et al., 1998, 2002; Niklas & Schneider, 2012), whereas other studies have found no significant differences between genders (Arens et al., 2016; Herbert & Stipek, 2005; Marsh et al., 2002). Other influencing factors include prior experiences with literacy, interest or motivation, and most importantly, precursors of literacy skills (Dapp & Roebers, 2018), i.e., ELS.

ELS refer to the skills, behavior, and knowledge linked to later reading and writing skills (Whitehurst & Lonigan, 1998). These skills encompass children’s understanding of concepts and functions of literacy, writing and composing, and their knowledge about letters and words (Sénéchal et al., 2001). As children are constantly exposed to language and literacy-related cues from an early age, they develop literacy-related skills even before they formally learn to read or write (Sénéchal et al., 2001). Thus, literacy acquisition begins early in children’s lives and is conceptualized as the development of ELS into reading and writing skills (Whitehurst & Lonigan, 1998).

The relation between ELS and reading and writing skills is inherent in their definition and the nature of literacy development. Accordingly, robust empirical evidence suggests that ELS develop into reading and writing skills (Claessens et al., 2009; Niklas & Schneider, 2017; Suggate et al., 2018). Beyond their role in literacy development, ELS also positively predict achievement in other academic domains such as mathematics (e.g., Pinto et al., 2016), and are associated with non-academic factors such as socio-emotional skills (e.g., Wirth et al., 2022).

### The relation between early literacy skills and literacy self-concept

The relation between ELS and LSC originates in the complex relation between academic self-concept and achievement and has been extensively studied, both cross-sectionally and longitudinally, considering both causal directions, from ELS to LSC and vice versa (see Hansford & Hattie, 1982; Huang, 2011; Möller et al., 2009, 2020; Valentine et al., 2004; Wu et al., 2021 for meta-analyses). There is consensus that these two variables are positively related, and medium to large effect size correlations are found for older children. However, the causal order of the two variables remains a subject of ongoing discussion, especially regarding young children (Guay et al., 2003).

Calsyn and Kenny (1977) laid the theoretical groundwork for this discussion by developing self-enhancement and skill development models. According to the self-enhancement model, a positive self-concept contributes to improved academic achievement, for example, by enhancing engagement and effort in domain-related activities (Retelsdorf et al., 2014). The skill development model, on the other hand, posits that academic achievement is the primary determinant of self-concept (Huang, 2011). Individuals evaluate their abilities based on social comparisons and past academic experiences, such as grades or feedback (Segerer et al., 2021). This evaluation then shapes their perception of competence in a particular academic domain (Locher et al., 2021).

Furthermore, efforts have been made to integrate these two models, as outlined in the Reciprocal Effects Model (Marsh & Craven, 2006). According to this model, academic self-concept and achievement are reciprocally related, meaning they are both a cause and an effect of each other (Möller et al., 2011). Recent longitudinal studies in various academic domains have substantiated the bidirectional relation between self-concept and achievement for children over eight years (Arens et al., 2017, 2022; Marsh et al., 2023; Preckel et al., 2013, 2017; Retelsdorf et al., 2014), including the domain of literacy (Chen et al., 2013; Guay et al., 2003; Möller et al., 2014; Niepel et al., 2014; Retelsdorf et al., 2014; Sewasew & Koester, 2019; Walgermo et al., 2018). Yet the onset of this relation has received limited investigation and remains debated (Dapp & Roebers, 2018). Although existing studies point to reciprocal relations between ELS and LSC during preschool (Chapman et al., 2000; Dapp & Roebers, 2018), it is still poorly understood when these relations emerge and which aspects play a role in their development. Consequently, it is important to consider factors such as the home learning environment that may contribute to shaping the relation between achievement and self-concept in preschool and a greater accuracy of self-concept.

### The role of the home literacy environment for an accurate literacy self-concept

In general, developing a positive self-concept is a highly valued educational goal, given it is positively associated with various academic outcomes, including interest and achievement (Marsh et al., 2005). However, having a positive self-concept alone does not guarantee improved future achievement and outcomes. Instead, it is the realistic and accurate self-concept that allows individuals to better assess their skills and have more engaged and motivated learning experiences (Sáinz & Upadyaya, 2016). When individuals accurately evaluate themselves, in alignment with their actual skills, they are more likely to make informed decisions that positively influence their life outcomes (Dunning et al., 2004).

However, the benefits of an accurate self-concept for preschool children are controversial. Young children often start tasks as beginners and do not perform at expert levels initially (Shin et al., 2007). Those who are highly aware of their limitations can therefore become discouraged quickly and switch tasks, while children who overestimate their abilities may persist longer and improve through practice. Consequently, their exaggerated (and unrealistic) predictions may serve as a buffer for their self-esteem and foster educational outcomes (Shin et al., 2007). Given these different perspectives, it is worth investigating how accurate young children’s self-concepts are and which factors influence the accuracy of their self-concept (Chiu & Klassen, 2009).

In this context, the family plays a key role: as German preschools usually do not provide formal instruction, early learning occurs primarily through parent–child interactions at home (Arens et al., 2016). Although these early interactions are typically informal and parental feedback is unsystematic, they help children develop a foundational self-understanding and are essential for self-concept formation (Thompson, 1998). Research suggests that early parent–child interactions such as sensitive caregiving, responsiveness, and positive communication, provide critical support for children’s cognitive development, indirectly fostering self-concept development (Paulus et al., 2018) and contributing to the early differentiation of skills across different domains (Dapp & Roebers, 2018).

The interactions within the family context related to literacy constitute what is known as the HLE (Niklas, 2015). The HLE refers to the activities and resources at home that support the development of children’s literacy skills (Niklas, 2015; Sénéchal & LeFevre, 2002). A high-quality HLE is characterized by frequent and meaningful shared reading experiences, access to a variety of age-appropriate books, rich verbal interactions between parents and children, and parental engagement in literacy-related activities (Mol & Bus, 2011; Sénéchal & LeFevre, 2002). The quality of the HLE has a strong positive impact on children’s ELS and later reading and writing skills (Foster et al., 2005; Niklas & Schneider, 2013, 2017; Nuswantara et al., 2022; Roberts et al., 2005; Weigel et al., 2006; Wirth et al., 2022). Children living in a high-quality HLE frequently engage with literacy and have opportunities to apply and observe their literacy skills at home (Sénéchal & LeFevre, 2014). Even though young children struggle with remembering information about themselves (Xia et al., 2022), a supportive learning environment may contribute to a better self-assessment (Yan et al., 2023): A high-quality HLE can foster important meta-cognitive skills (Dearing et al., 2012; Whitebread et al., 2009), including meta-cognitive knowledge (knowledge about task, strategy, and personal factors influencing cognitive performance) and meta-cognitive regulation (planning, monitoring, controlling, and evaluating one’s own learning). For instance, when parents engage in shared reading or scaffolded problem-solving, they frequently prompt children to reflect on their own learning processes and accuracy. Over time, these interactions help children calibrate their self-assessments. Furthermore, children living in a high-quality HLE receive more frequent high-quality feedback and have a greater variety of opportunities to test their skills and thus evaluate their LSC, whereas children from a lower-quality HLE have fewer opportunities and thus lack sufficient evidence on which to base their LSC. As a result, children living in a high-quality HLE may be able to better estimate their skills and develop a more accurate LSC that more closely corresponds to their actual ELS level compared to children living in a lower-quality HLE.

The accuracy of children’s LSC, i.e., how closely LSC is related to children’s ELS, can also be explained through the lens of the Dunning-Kruger effect. This effect suggests that strong performers are better at estimating their skills, whereas poor performers often perceive themselves as more competent than they are in reality (Dunning, 2011; Kruger & Dunning, 1999). Applying this to the HLE, children living in a high-quality HLE are typically the ones who perform better and who are able to make more accurate judgments about their competence levels. In contrast, children in a lower-quality home learning environment often demonstrate lower metacognitive abilities, which are necessary for accurate self-assessment, and may not be able to develop accurate self-concepts at this age (Kruger & Dunning, 1999).

However, evidence on the association of the HLE with children’s LSC is lacking, although some studies show positive relations between the home learning environment and self-concept (Crampton & Hall, 2017; Sammons et al., 2016). Research examining the influence of the family on the accuracy of LSC also remains limited, with most studies focusing on family background variables such as culture or socioeconomic status (Chiu & Klassen, 2009; Sewasew & Koester, 2019). Even though these variables are closely linked to the HLE, none of these studies have focused directly on the HLE. The lack of research in this context is surprising, considering that several studies have recommended investigating how the HLE might impact the relation between ELS and LSC as a direction for future research (Crampton & Hall, 2017; Katzir et al., 2009; Liu et al., 2018). Furthermore, none of these studies have targeted the preschool age group.

## Present study

As research has delivered mixed findings regarding the relation between ELS and LSC, this study aims to investigate this complex relation in preschool children. It also goes beyond the existing work by adding the role of HLE in the relation between ELS and LSC. As a first step, we address the research gap in the rank-order stability of the constructs of LSC and ELS and the relation between them. These primary investigations are conducted before evaluating the main research question (i.e., the role of the HLE):

- H1: Based on prior research, we hypothesize that the ELS (H1a) and LSC (H1b) of preschool children would remain stable across a period of two years (Arens et al., 2016; Suggate et al., 2018), understood as rank-order stability of interindividual differences over time.
- H2: Further, we expect positive correlations between ELS and LSC both at the same time point and across different time points, as suggested by previous studies (Chapman et al., 2000; Dapp & Roebers, 2018).

As the main focus of the study, we further investigate the relation of HLE with both ELS and LSC and its moderating role in the relation between them:

- H3: We assume that the HLE would be positively associated with both ELS (H3a) (e.g., Foster et al., 2005; Niklas & Schneider, 2017) and LSC (H3b) (e.g., Crampton & Hall, 2017; Sammons et al., 2016).
- H4: The HLE is also expected to contribute to the accuracy of LSC by moderating the relation between ELS and LSC. To tackle the research gap in this context, we exploratively examine whether children with a higher-quality HLE have a more accurate LSC than children with a lower-quality HLE.

## Methods

### Sample and procedure

This study was carried out as part of the large-scale longitudinal project “Learning4Kids”, conducted between June 2020 and August 2022 in Germany (see Niklas et al., 2020, 2022, for study protocols). Learning4Kids had a two-cohort study design and included comprehensive assessments of children’s skills and family backgrounds. Participants were recruited via preschools and the Government Department administration of Munich. Participation in the study was voluntary and contingent upon parental consent. The study received ethical approval from the University of Munich (LMU).

We used data from the first four time points (t1-t4) of both cohorts. The total sample consisted of 500 preschool children with a mean age of 61 months (*SD* = 4.6, min = 51, max = 75 months) at t1 (penultimate year of preschool/kindergarten). Altogether, 51.4% of the children in the sample were girls, and 42% had a migration background.

Assessments were conducted with children and their families every five to six months. They took place in the homes of participating families. During these two-hour visits at home, trained experimenters assessed children’s ELS and LSC, among other preschool skills. Meanwhile, parents completed a comprehensive paper–pencil questionnaire, which included questions on the HLE and other family and child background variables. All assessments with children were conducted in German, which serves as the official instructional language in the school system. Nevertheless, to ensure that language barriers did not confound the results and that all children fully understood the tasks, the assessments utilized play-based tests that relied on minimal and simplified verbal instructions. Moreover, example items ensured that children understood each task before the test items were assessed. Twelve language options (most spoken languages in Germany, e.g., Turkish, Russian) were available for the parental questionnaire.

### Measures

#### Early literacy skills

ELS of children comprised their active and passive vocabulary skills, phonological awareness, and letter knowledge, which were assessed with six validated and standardized subtests at each time point.

In the first three time points (t1-t3), active vocabulary was measured using 15 items from the *Active Vocabulary Test* (AWST-R; Kiese-Himmel, 2005). At t4, the active vocabulary test was switched to the *Vocabulary and Word Finding Test* (WWT; Glück, 2011) due to ceiling effects. The reliability of the former was acceptable (Cronbach’s α_*t1-t3*_ =.79), while the reliability of the latter was good (Cronbach’s α =.82). The two tests showed high correlations of at least *r* =.70.

Passive vocabulary was assessed using the German version of the *Peabody Picture Vocabulary Test* (Lenhard et al., 2015) at all time points with nine sets of items (sets 4–12), each containing 12 items. The test revealed excellent reliability with Cronbach’s α_*t1-t4*_ between.93 and.94 (for full reliability indices of all measures, see Appendix, Table 3).

Children’s phonological awareness was assessed with two subtests from the *Würzburg Preschool Test* (WVT; Endlich et al., 2017). In the first subtest, children had to identify the word that did not rhyme with the others in a set of four (e.g., *Hose – Oma – Dose – Rose. Which word does not rhyme with the others?*). The second subtest focused on initial sound recognition, with children naming the initial sound of the word presented in a picture. Both subtests started with eight items, and three additional items were used at t3, as well as two more at t4 to prevent ceiling effects. The additional items were only presented if the child had made fewer than two errors in the first eight items. The reliability was at least acceptable for the rhyming test (Cronbach’s α_*t1-t4*_ between.76 and.82) and good for sound recognition (Cronbach’s α_*t1-t4*_ between.88 and.90).

Letter knowledge was assessed with two WVT subtests: receptive and productive letter knowledge. In the receptive letter knowledge test, the experimenters presented 10 letters to the children. The children were asked to identify the correct letter out of four letters shown by the experimenters, who named the target letter*.* In the productive letter knowledge subtest, children were asked to name 10 displayed letters. Both letter knowledge tests comprised three additional items at t3 and another two at t4. Both tests showed acceptable to good reliability (Cronbach’s α_*t1-t4*_ between.77 and.88 for receptive letter knowledge, and between.85 and.90 for productive letter knowledge).

For each ELS subtest, total scores were calculated and z-standardized to account for variable test lengths. The overall ELS score was the mean of the z-standardized total subtest scores. The overall ELS scale showed good reliability (Cronbach’s α_*t1-t4*_ =.80 to.81).

#### Home literacy environment

The HLE was assessed with 15 items in the parental questionnaire at all time points (see Appendix, Table 4). Parents reported their shared book reading, linguistic activities, literacy exposure, literacy instructions, and aspects of the formal HLE on a five-point Likert scale (0 = “less often/never” to 4 = “several times a day”). The HLE items were developed within the study based on previous instruments (Niklas & Schneider, 2013; Niklas et al., 2016a, 2016b). The items were z-standardized, and the mean of the z-standardized HLE items constituted the overall HLE score. The reliability of the overall HLE scale was acceptable to good, with Cronbach’s α ranging from.76 to.81.

#### Literacy self-concept

LSC was measured using the validated LSC scale of the *Pictorial Scale for the Assessment of Enjoyment of Learning and Self-Concept of Preschool Children in Mathematics and Literacy* (Roux et al., 2010). During the assessments, children were shown two pictures of a child of their gender and told that one child is good at a particular skill, while the other is not as good at it. The children then indicated which child they felt they resembled more. They were further asked to specify if they were (4) *very good* or (3) *quite good*, or if their skills were (2) *quite lacking* or (1) *very lacking*. The test included seven items: writing, reading, distinguishing between different alphabets, making up stories, recognizing letters, rhyming, and making up words. The reliability of the composite scale was acceptable (Cronbach’s α_*t1/t2/t3/t4*_ =.77/.75/.67/.70).

#### Covariates

We considered some relevant child characteristics and family background variables in the analyses, such as children’s age and gender (e.g., Dapp & Roebers, 2018; Marsh & Yeung, 1998). We also controlled for children’s (nonverbal) intelligence as an important predictor of their self-concept and potentially influencing the alignment between self-concept and actual performance (Gose et al., 1980). Measuring nonverbal intelligence is particularly crucial here because it assesses cognitive functioning independently of language skills, ensuring that differences in verbal ability or language proficiency do not confound the measure of general intelligence. Nonverbal intelligence was assessed at t1 using the *Columbia Mental Maturity Scale* (Burgemeister et al., 1972). In this entirely nonverbal visual classification test, children were presented with a series of pictures and had to identify which object out of four did not belong to the others. Additionally, we accounted for migration background, which is particularly relevant for language-related skills (Valcárcel Jiménez et al., 2024). Children were classified as having a migration background if they or at least one of their parents was not born in Germany. The socioeconomic status of the families was not considered, as it was expected to be highly associated with migration background in a German sample (Niklas & Schneider, 2017).

### Data analysis

Statistical analyses were performed with R.4.2.1 (R Core Team, 2022). Before the analyses, manifest variables of ELS, LSC, and HLE for each time point were created. For the primary analyses, missing values were omitted by default, and only complete observations were used. We conducted a Little’s MCAR (missingness completely at random) test, which resulted in a significant finding (*p* <.001). Looking closer at the individual variables, data of LSC at t1 were missing to a greater extent for younger children, while data on HLE at t1 were more likely to be missing for children with lower baseline ELS. At t4, missingness in ELS was significantly greater in children with a higher baseline HLE and in children with a migration background. Missingness at t4 for both LSC and HLE was also greater for children with a migration background, suggesting that families with a migration background tended to drop out at t4. However, attrition analyses using *t*-tests revealed that children whose families dropped out of the study (*n* = 46) did not differ significantly from those who participated at all time points (*n* = 454) in their ELS, LSC, HLE at t1, or background variables (see Appendix, Table 5 for statistics).

As missingness was not completely at random, we also conducted our analyses with imputed data using the predictive mean matching method from the *mice* package (van Buuren & Groothuis-Oudshoorn, 2011). This resulted in two separate datasets for our analyses: the original complete-case dataset and the fully imputed dataset. The former was used for the main analyses, while the latter was used to validate the robustness of the findings, and we show both results for transparency.

With both datasets (original and imputed data), we conducted bivariate correlations between ELS and LSC at different time points to investigate the stability and associations of ELS and LSC from t1 to t4. We focused on statistically significant cross-time correlations, evaluating the degree of rank-order stability based on the size of the correlation coefficients. Furthermore, correlations between HLE and both ELS and LSC were analyzed, incorporating all time points for each variable. The correlation analyses served as a first insight into the relation between relevant study variables.

To investigate the main research questions, we conducted cross-sectional multiple linear regressions for each time point. While the dataset utilizes a longitudinal design, the primary objective of this study is to examine the moderating role of the HLE on the relation between ELS and LSC. To maintain a clear and targeted focus on these interaction effects, we opted for time-point-specific (and cross-sectional) analyses to establish a foundational understanding of these effects. ELS, HLE, gender, age, migration background, nonverbal intelligence, and the interaction term between ELS and HLE were used to predict LSC. ELS, HLE, and LSC values from the same time point were included in the analyses. As a further step, we conducted simple slopes analyses to investigate the relations between ELS and LSC at different levels of the HLE. We defined these levels using key values of the moderator (± 1 *SD* from its mean), resulting in three groups: low-quality HLE (−1 *SD* and below), average-quality HLE (−1 to + 1 *SD*), and high-quality HLE (+ 1 *SD* and above; Long, 2024).

Given that home language is a central factor in literacy development (Valcárcel Jiménez et al., 2024), we conducted supplementary analyses stratified by migration background. Although our operationalization of migration background does not directly capture the spoken language at home, those two constructs are closely related and the majority of children with a migration background speaks a language other than German at home (Autorengruppe Bildungsberichterstattung, 2016). To formally test whether the findings of the regressions differed between children with and without a migration background, we extended our main models to include further two-way (e.g., ELS x Migration Background) and three-way interactions (ELS × HLE × Migration Background). As these analyses were exploratory and fell outside our primary hypotheses, the results are provided in the Appendix for further interested readers.

## Results

Table 1 presents correlations among all variables of interest in the original dataset (see Appendix, Table 6 for sample sizes). ELS were strongly intercorrelated across different time points (H1a; *r* between.75 and.85). The correlations in LSC across different time points were also consistently significant, with weak to moderate correlations (H1b; *r* between.27 and.39). LSC of children at t1 was negatively correlated with their ELS at t2-t4. LSC at t2 and t3 did not show significant correlations with ELS at any time point. The correlations between LSC at t4 and ELS at all time points were significant and positive (H2: *r* between.10 and.22). Moreover, ELS showed significant positive correlations with HLE at all timepoints (H3a; *r* between.26 and.41). In contrast, the only significant correlations between the HLE and LSC was found for HLE t2 and LSC t4 (H3b).

**Table 1:** Correlations of all variables of interest

	1	2	3	4	5	6	7	8	9	10	11	12	13	14	15
1. ELS t1															
2. ELS t2	.85^***^														
3. ELS t3	.80^***^	.85^***^													
4. ELS t4	.75^***^	.80^***^	.82^***^												
5. HLE t1	.41^***^	.38^***^	.40^***^	.32^***^											
6. HLE t2	.38^***^	.39^***^	.41^***^	.33^***^	.77^***^										
7. HLE t3	.28^***^	.27^***^	.30^***^	.22^***^	.74^***^	.72^***^									
8. HLE t4	.26^***^	.28^***^	.28^***^	.23^***^	.69^***^	.72^***^	.72^***^								
9. LSC t1	-.06	-.11^*^	-.12^**^	-.11^*^	-.01	-.02	-.02	-.01							
10. LSC t2	-.01	-.04	-.05	-.02	.04	-.00	.02	.01	.38^***^						
11. LSC t3	.03	.00	.05	.07	.07	.03	.08	.01	.32^***^	.39^***^					
12. LSC t4	.10^*^	.10^*^	.12^**^	.22^***^	.04	.10^*^	-.08	.04	.27^***^	.30^***^	.37^***^				
13. Age	.12^**^	.14^**^	.07	.26^***^	-.21^***^	-.12^*^	-.16^***^	-.12^*^	.12^*^	.04	-.02	.26^***^			
14. Gender	.11^*^	.09	.06	.00	.09^*^	.11^*^	.03	.03	.05	.04	-.04	-.00	-.05		
15. MB	-.38^***^	-.37^***^	-.36^***^	-.31^***^	-.25^***^	-.24^***^	-.19^***^	-.24^***^	.12^**^	.08	.03	.05	.04	-.02	
16. NI	.22^***^	.28^***^	.26^***^	.28^***^	.11^*^	.15^**^	.07	.11^*^	-.14^**^	-.08	-.06	.13^**^	.15^***^	.13^**^	-.06

*ELS* = Early Literacy Skills. *HLE* = Home Literacy Environment. *LSC* = Literacy Self-Concept. *MB* = Migration Background. *NI* = Nonverbal Intelligence. ^*^p <.05. ^**^p <.01. ^***^p <.001.

To investigate the main research question, multiple linear regressions with the interaction term between ELS and HLE were used to predict LSC (H4). The relevant findings of the regression analyses are shown in Table 2 (see Appendix, Table 7 for full results).

**Table 2:** Cross-sectional multiple linear regressions with the interaction term between early literacy skills and home literacy environment to predict literacy self-concept

	*n*	β	*SE*	*t*	*p*	Adjusted* R*^*2*^
t1	455					.04
ELS t1		-.01	.08	−0.15	.879	
HLE t1		.13	.11	1.22	.222	
ELS t1*HLE t1		.06	.12	0.53	.594	
t2	458					.01
ELS t2		.04	.08	0.53	.598	
HLE t2		.11	.11	1.03	.305	
ELS t2*HLE t2		.35	.13	2.75	.006	
t3	455					.01
ELS t3		.09	.08	1.25	.213	
HLE t3		.21	.10	2.06	.040	
ELS t3*HLE t3		.25	.12	1.98	.048	
t4	434					.11
ELS t4		.27	.07	3.66	<.001	
HLE t4		.17	.11	1.60	.109	
ELS t4*HLE t4		.31	.12	2.63	.009	

*ELS* = Early Literacy Skills. *HLE* = Home Literacy Environment. ELS*HLE = the interaction term between ELS and HLE. In the regression analyses, standardized ELS, HLE, and LSC scores were used, and gender, age, migration background, and nonverbal intelligence were controlled for at all time points. Only complete observations were used.

At t1, only age (β =.15, *SE* =.05, *p* =.002), migration background (β =.10, *SE* =.05, *p* =.038) and nonverbal intelligence (β = -.15, *SE* =.05, *p* =.002) significantly predicted LSC. Simple slopes analyses further indicated that ELS were not significantly associated with LSC (*p* >.05) for any group of children (low-quality HLE, average HLE, and high-quality HLE).

**Fig. 1:**
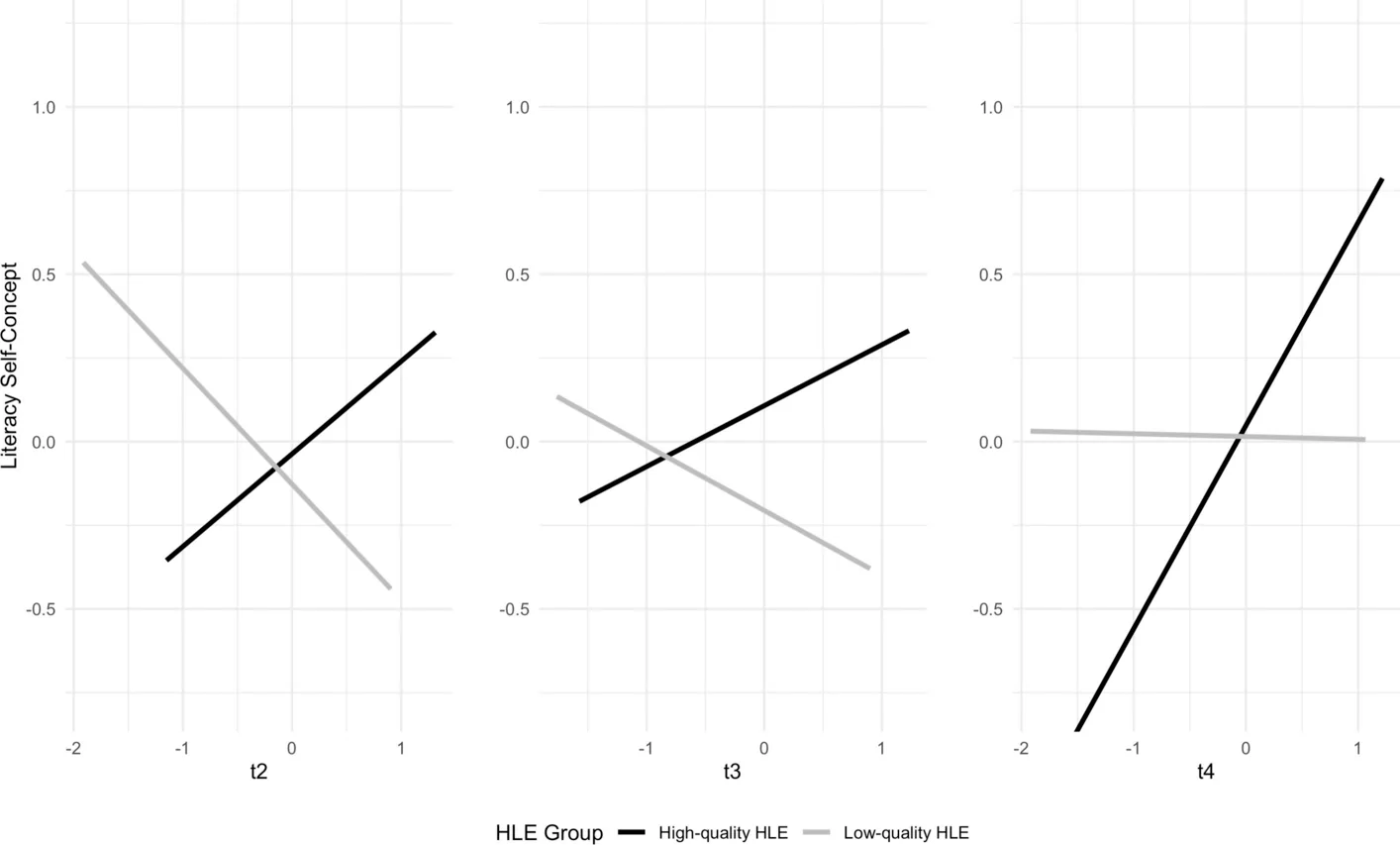
Relation between early literacy skills and literacy self-concept for children with a high-quality and a low-quality home literacy environment. Note. HLE = Home Literacy Environment. HLE groups defined as 1 *SD* above (high-quality HLE) and 1 *SD* below the mean (low-quality HLE). Literacy self-concept was predicted by early literacy skills, HLE, age, gender, migration background, and nonverbal intelligence.

At t2, only the interaction term between ELS and HLE was a significant predictor of LSC (β =.31, *SE* =.13,* p* =.006). The visual investigation of the interaction at t2 showed that ELS positively predicted LSC for children with a high-quality HLE and negatively for children with a low-quality HLE (see Fig. 1, left). Children from a low-quality HLE rated their literacy skills higher when they actually had lower ELS and, in contrast, the LSC of children from a higher-quality HLE aligned with their ELS. This was confirmed by the simple slopes analysis for the high-quality HLE group (1 *SD* above the mean; β =.23, *SE* =.10, *p* =.003), but not for the low-quality HLE group (β = -.13, *SE* =.10, *p* =.22).

At t3, the HLE (β =.21, *SE* =.10,* p* =.040) and the interaction term between HLE and ELS (β =.25, *SE* =.12, *p* =.048) significantly predicted LSC. The relation between ELS and LSC was again only significant and positive for children with a high-quality HLE (β =.22, *SE* =.10, *p* =.003; see also Fig. 1, middle).

At t4, ELS (β =.28, *SE* =.07, *p* <.001), age (β =.19, *SE* =.05, *p* <.001), migration background (β =.12, *SE* =.05, *p* =.014), and the interaction between HLE and ELS (β =.30, *SE* =.12,* p* =.012) were significant predictors for LSC. Here, ELS were more closely associated with LSC for children with a high-quality HLE than for those with a low-quality HLE, according to a visual investigation (see Fig. 1, right) as well as a simple slopes analysis. The relation between ELS and LSC was significant for children both with an average HLE (β =.28, *SE* =.07, *p* <.001) and a high-quality HLE (β =.42, *SE* =.10, *p* <.001), but not significant for those with a low-quality HLE (β =.13, *SE* =.09, *p* =.14).

The reported analyses were further conducted using the imputed dataset, yielding largely consistent results with the original data for correlational analyses. The significance of the correlations between the variables of interest remained unchanged (see Appendix, Table 8), except for the emerging significant correlation between HLE and LSC at t4 (*r* =.12, *p* =.008). However, some differences emerged in the regression analyses (see Appendix, Table 9). The interaction term between ELS and HLE, which had previously shown significance, no longer significantly predicted LSC at t2 or t3 (t2: β =.21, *SE* =.12, *p* =.077; t3: β =.15, *SE* =.12, *p* =.231), although the *p*-value was only slightly above the threshold at t2. Consequently, the interaction term was only significant at t4 (β =.32, *SE* =.14, *p* =.025), suggesting that the relation between ELS and LSC was stronger for children with a higher HLE than for those with a lower HLE.

## Discussion

This study examined the associations between ELS and LSC in preschool children across different time points and the role of the HLE in this relation. While both constructs showed significant positive auto-correlations over time, cross-sectional correlations between ELS and LSC at the same time point only emerged at t4. LSC at t1 was negatively associated with ELS at all time points, whereas LSC at t4 was positively associated with ELS at all time points. HLE was consistently positively related to ELS across all time points, but showed no significant association with LSC. Moderation analyses revealed that the interaction between ELS and HLE significantly predicted LSC at t2 through t4, indicating that a higher-quality HLE was associated with a stronger positive relation between ELS and LSC. However, a significant interaction effect could only be validated for t4 in the imputed data.

In our first hypothesis (H1), we proposed that both ELS and LSC would demonstrate rank-order stability over time, which was supported by the findings of this study. ELS demonstrated significant rank-order stability across all time points with strong correlations both in the original and imputed data, aligning with previous studies (Claessens et al., 2009; Niklas & Schneider, 2017; Suggate et al., 2018). Similarly, LSC remained stable in its relative rank order over time; however, with weak to moderate correlations. Previous research has also shown a stable self-concept during the preschool period (Arens et al., 2016; Verschueren et al., 2012), including a focus on LSC (Arens et al., 2016; Marsh et al., 1998). This indicates that while children’s LSCs may undergo some changes during the preschool years, their relative tendencies towards a positive or negative LSC remain similar over time.

The relation between ELS and LSC (H2) showed inconsistent findings across different time points. Children who had a higher LSC at t1 showed significantly lower ELS at later points, while those with higher ELS at any time point had a higher LSC at t4. The negative relations between LSC at t1 and ELS hint at a contradiction to self-enhancement models (Calsyn & Kenny, 1977), but also align with previous research (Cohrssen et al., 2016). These findings suggest that young children may not yet be able to base their LSCs on their actual abilities, possibly because they do not receive clear instructions and feedback regarding these abilities. In fact, children with a higher self-concept may even exhibit lower skills because they maintain positive self-expectations to protect their self-esteem (Shin et al., 2007).

Our findings contradict previous studies suggesting realistic self-concepts even at younger ages (Chapman et al., 2000; Dapp & Roebers, 2018), while supporting Shavelson’s view that preschool children are not yet capable of developing accurate self-concepts (Shavelson et al., 1976, see also Cohrssen et al., 2016). Furthermore, the findings revealed positive relations between ELS and LSC at t4. These results suggest that objective ability levels and achievement become more important, and LSC tends to align with literacy skills upon school entry (Möller et al., 2009). Previous research has also shown that older children tend to develop more accurate self-concepts, as their experience with literacy followed by feedback increases (Chen et al., 2013; Guay et al., 2003; Möller et al., 2014; Niepel et al., 2014; Retelsdorf et al., 2014; Sewasew & Koester, 2019; Walgermo et al., 2018).

Interestingly, supplemental analyses revealed that the negative correlations between ELS and LSC were not present among children with a migration background (see Appendix, Table 10). Instead, their ELS at t1 was already positively associated with their self-concept at t2, and bidirectional positive correlations between ELS and LSC continued until t4. In contrast, children without a migration background showed negative associations at t1 and partly at t2, with the only positive correlation appearing cross-sectionally at t4.

This contradicts prior research demonstrating that children with a migration background may have more biased self-concepts and overestimate their abilities (Segerer et al., 2021), suggesting the opposite: children without a migration background showed inaccurate self-concepts, while those with a migration background held a rather accurate one. A key explanation for this discrepancy may be children’s age. The study by Segerer et al. (2021) was conducted with primary school students, whose self-concepts are increasingly more accurate than those of younger children. In our preschool sample, where overestimation is generally common, children with a migration background may diverge for a specific reason: due to language barriers, these children might perceive their challenges regarding German literacy more acutely, leading them to rate their skills more realistically or critically than their peers.

Moreover, consistent with prior studies (Hood et al., 2008; Sénéchal & LeFevre, 2014) and our hypothesis (H3a), the HLE showed positive correlations with ELS across all time points. While our findings are based on correlational data, they are in line with experimental and longitudinal studies suggesting that the HLE plays an important role in the development of ELS (Foster et al., 2005; Niklas & Schneider, 2013, 2017; Nuswantara et al., 2022; Roberts et al., 2005; Weigel et al., 2006; Wirth et al., 2022). The results also show that the HLE is not linked to LSC, except for the small positive correlation between HLE at t2 and LSC at t4, contradicting the findings from studies involving older children (Crampton & Hall, 2017; Sammons et al., 2016) and H3b. However, this could indicate that a potential impact of HLE on LSC only becomes apparent in later years.

Regarding our main hypothesis (H4), the interaction between ELS and HLE significantly predicted LSC from t2 to t4. Specifically, children with a higher-quality HLE demonstrated a stronger positive relation between their ELS and LSC than those with a lower-quality HLE. Although the causal direction of this relation cannot be determined from cross-sectional analyses alone, the findings align with the expectation that a high-quality HLE may enhance children’s ability to accurately assess their literacy skills, supporting the idea that the HLE contributes to a more accurate LSC. However, the interaction term was no longer a significant predictor of LSC at t2 and t3 when the imputed data were used, with a significant prediction only found at t4. Although the significance of the interaction effects differed between the original and imputed datasets, the overall trend remained consistent. The role of the HLE in the relation between ELS and LSC tended to emerge predominantly at later stages of children’s development.

Hypothesis 4 was exploratory and could not be based on previous findings, yet it provides important first evidence regarding the role of the HLE in an accurate self-concept in young children. The finding that children with a higher-quality HLE showed more accurate LSCs aligns with the Dunning-Kruger effect (Kruger & Dunning, 1999). However, unlike this effect, which posits that low performers tend to overestimate their abilities, we found that children with better ELS living in a low-quality HLE may underestimate their LSC compared to their peers. It is also important to note that the Dunning-Kruger effect is based on adults and may not be exactly applicable to young children. These results indicate that children in low-quality HLEs may not receive adequate feedback and reinforcement at home, leading to inaccurate LSCs. This may be particularly true for children from disadvantaged backgrounds, i.e., children from low socio-economic households, who often experience lower-quality HLEs. Even when these children demonstrate relatively strong skills, they may underestimate their skills, potentially due to negative feedback from others. Sociological and psychological frameworks suggest that young children’s self-evaluations are mainly formed by internalizing the perceived appraisals of significant social figures such as parents or teachers (Mead, 1934). When children encounter lower expectations or biased feedback, they may internalize these negative external signals (e.g., Evans et al., 2011). This can lead them to doubt or undervalue their objective abilities, resulting in a deflated self-concept. For children with a migration background in particular, the HLE does not seem to play a large role in the accuracy of LSC (see Appendix, Table 11). Rather, these children tended to show associations between their ELS and LSC from a certain age, regardless of the HLE, although the moderating role of the HLE did not significantly differ between children with and without a migration background (see Appendix, Table 12). In this regard, our findings provide important insights into the extent to which HLE is associated with children’s ability to accurately assess their own skills and raise the important question of what distinguishes children who are able to do so from those who are not. At the same time, it is important to recognize that highly accurate self-assessments may not necessarily be the optimal developmental outcome in early childhood. A positive self-concept or slight overestimation of one’s abilities may foster motivation, persistence, and willingness to engage with challenging tasks (Marsh et al., 2005; Shin et al., 2007), whereas a strongly unrealistic self-concept may hinder adaptive learning (Sáinz & Upadyaya, 2016). Therefore, it is important to examine the conditions under which children may develop positive yet realistic self-concepts.

### Limitations and further research

While the findings shed light on important relations, it is also crucial to acknowledge certain limitations. An important limitation is comparability issues due to changes in the ELS measurement, as we used additional items to assess phonological awareness and letter knowledge at the last two measurement points. There was also a switch in the active vocabulary test at t4. However, these changes were necessary to eliminate ceiling effects and to conduct age-appropriate assessments of the ELS.

Another limitation of the study is the potential role of social desirability in the HLE assessment, as self-reports were used to assess the HLE. Previous research, however, has demonstrated strong associations between self-reports and other measures of HLE, such as children’s literature knowledge or diary entries (Burgess, 2002). Nevertheless, future research could also incorporate more objective measures for capturing HLE, as standardized checklists and observational methods additional to self-reports can provide a more comprehensive and less biased assessment of the HLE (e.g., Grolig et al., 2017; Wirth et al., 2023). As the operationalization of the HLE in this study focused on quantitative aspects of the HLE (e.g., frequency of activities or variety of resources), future research could also incorporate the quality of parent–child interactions within the HLE as important predictors of children’s literacy skills (Valcárcel Jiménez et al., 2026). Such assessments could go beyond literacy activities and examine important parent–child interaction qualities such as parental sensitivity or responsiveness (Paulus et al., 2018), and may also include parental views of children’s abilities.

Moreover, the analyses were only correlational and cross-sectional, which cannot account for the temporal ordering of the causal relation between ELS and LSC. In this study, LSC was selected as the outcome variable because we aimed to explore self-concept development in young children rather than the acquisition of ELS. Besides, the focus on cross-sectional data was intentional, as this study aimed to explore the relations as an initial step. Still, future longitudinal research, which was outside the scope of this paper, is needed to investigate the influence of the HLE on the accuracy of LSC over time and to unravel the causal mechanisms underlying these associations. Such analyses can also provide insights into which quality aspects of the HLE are associated with more vs. less accurate judgements in young children.

The validation of the results using imputed data revealed some differences, particularly in the regressions. The significant interaction effects between ELS and HLE could not be observed at t2 and t3, limiting the robustness of the findings. Therefore, while the moderating role of HLE in the relation between ELS and LSC was observed in both datasets, the developmental stage at which this effect emerges remains unclear. Nevertheless, the observed trends were consistent across both datasets. Still, given these limitations and the exploratory nature of the study, replications are crucial to confirm the robustness and generalizability of the findings.

Focusing on the most important finding of this study, the interaction effects, future studies could explore in greater detail which specific aspects of the HLE support the development of a positive LSC and which may not, for example, with a Latent Profile Analysis. The finding that children with strong skills may underestimate themselves in low-quality HLEs underscores the importance of the HLE’s feedback function. This could be further examined within the framework of Deci and Ryan’s (2008) self-determination theory: perhaps children at this age still rely on motivating and supportive feedback, which may be more present in high-quality HLEs, to develop an accurate and positive LSC. Overall, further analyses are needed to clarify what constitutes a high-quality HLE and how its components contribute to children’s self-concept development.

### Strengths and implications

Our study analyzed data from a large sample of preschool children and provides insights into children’s LSC in preschool in relation to their ELS and the potential role of the HLE for an accurate LSC. This study addresses a significant research gap by focusing on preschool children, a group that has been underrepresented in self-concept research. Additionally, it is the first study to investigate the role of the HLE in the relation between ELS and LSC, providing valuable new insights into early childhood self-concept.

The findings of this study also carry important implications for educational practices. The demonstrated associations between LSC and ELS over time suggest that early childhood is a critical period for shaping both children’s self-perceptions and academic competencies. Parents and educators should focus on creating rich literacy environments and providing consistent, constructive feedback from the earliest stages of development. They should be aware that young children are already forming ideas about their abilities, and these perceptions can influence motivation, engagement, and learning trajectories over time. Furthermore, the stigma that self-evaluations in early childhood are too immature or random to be meaningful should be reconsidered.

Early interventions should also aim to enhance the HLE from an early age, as it is closely related to children’s literacy skills and contributes to a more accurate yet positive self-concept. Given the strong link between HLE quality and early learning outcomes, preschools and early education programs should consider integrating targeted initiatives that combine literacy-rich activities with strategies designed to foster self-concept. Such programs are especially important for children from disadvantaged backgrounds or low-quality HLEs, who may otherwise lack access to these foundational experiences. In short, the findings of this study highlight the importance of addressing the HLE early on as a means to promote educational equity and improve academic trajectories.

## Conclusion

This study shows the importance of early years in literacy skills and self-concept development. The HLE is closely associated with ELS of preschool children, but not with their LSC. However, children with a high-quality HLE have a more accurate and still positive LSC compared to children living in low-quality HLEs. Especially children with high ELS and low-quality HLE struggle to accurately estimate their skills and show a pattern of underestimation. These findings indicate that, although preschool children generally do not have accurate LSCs, parents may help to bridge the gap between self-concept and actual skills by offering high-quality literacy activities at home. By fostering a supportive and enriched HLE, parents can enhance their children’s ELS and facilitate the development of a positive and accurate LSC closely tied to their ELS.

## Data Availability

The data is available at: https://osf.io/6tegr/. Materials and code will be made available on request.

## Appendix

**Table 3:** Reliability results for all scales (Standardized Cronbach’s α)

	t1	t2	t3	t4
Literacy Self-Concept	.77	.75	.67	.70
Early Literacy Skills (ELS)				
*Active Vocabulary*	.79	.79	.79	.82
*Passive Vocabulary*	.93	.94	.94	.94
*Rhyming*	.77	.76	.82	.80
*Initial Sound Recognition*	.88	.88	.90	.90
*Receptive Letter Knowledge*	.77	.81	.86	.88
*Productive Letter Knowledge*	.87	.85	.87	.90
*Overall ELS*	.80	.81	.81	.80
Home Literacy Environment	.81	.80	.80	.76

**Table 4:** Operationalization of the home literacy environment

Question/Statement	Response Categories
How often do you and your partner read to your child at the moment?	*several times a day – daily – several times a day – once a week – less often/never*
Does your often child read alone or look at picture books by himself/herself?	*several times a day – daily – several times a day – once a week – less often/never*
How many books, approximately, do you own in your household? (Everything included, also nonfiction and cooking books, but no e-books)	*none – 1–10–11–50–51–100–101–150–151–200 –* > *200 books*
How many picture or pixie books are there in your household?	*none – 1–5 – 6–10–11–20–21–50–51–100 –* > *100 books*
How often do you visit libraries/public bookstores with your child at the moment?	*several times a week – weekly – approx. once a month – less often – never*
How often do you sing or recite poems or rhymes with your child?	*several times a day – daily – several times a day – once a week – less often/never*
To what extent do you agree with the following statement: “We like reading in our family.”?	*very strongly – strongly – slightly – to a lesser extent – not at all*
To what extent do you agree with the following statement: “In our family, we often talk about things we’ve read.”?	*very strongly – strongly – slightly – to a lesser extent – not at all*
To what extent do you agree with the following statement: “My child is very interested to read him/herself to and is looking forward to it.”?	*very strongly – strongly – slightly – to a lesser extent – not at all*
To what extent do you agree: “Reading is an important activity in our family.”?	*very strongly – strongly – slightly – to a lesser extent – not at all*
At home, I often explain to my child that we read from left to right, what a word or a sentence is and where a sentence starts/ends.	*does not apply at all – does rather not apply – neither wrong nor right – does apply a little – does exactly apply*
At home, I teach my child specific letters and how to write them.	*does not apply at all – does rather not apply – neither wrong nor right – does apply a little – does exactly apply*
At home, I practice the alphabet with my child, e. g., by having them recite or sing it.	*does not apply at all – does rather not apply – neither wrong nor right – does apply a little – does exactly apply*
At home, I encourage my child to recognize or read simple words, letters and words, e. g. their own name or the name of their favorite animal.	*does not apply at all – does rather not apply – neither wrong nor right – does apply a little – does exactly apply*
At home, we memorize poems or rhymes or songs.	*does not apply at all – does rather not apply – neither wrong nor right – does apply a little – does exactly apply*

**Table 5:** Attrition analysis

	*t*	*df*	*p*
Early literacy skills	0.76	52.83	.450
Home literacy environment	-1.00	47.86	.322
Literacy self-concept	0.32	51.55	.752
Age	0.11	54.18	.910
Gender	1.44	54.61	.154
Migration background	-1.56	52.68	.125
Nonverbal intelligence	1.36	53.62	.179

*t*-tests compared children who dropped out from the study and those who did not

**Table 6:** Sample sizes for the correlations

	1	2	3	4	5	6	7	8	9	10	11	12	13	14	15	16
1. ELS t1																
2. ELS t2	482															
3. ELS t3	478	482														
4. ELS t4	444	447	451													
5. HLE t1	459	458	456	425												
6. HLE t2	456	460	459	426	440											
7. HLE t3	454	458	460	429	430	440										
8. HLE t4	430	436	435	433	413	414	418									
9. LSC t1	487	487	483	448	463	461	459	433								
10. LSC t2	484	488	486	451	462	464	462	436	489							
11. LSC t3	475	479	483	447	454	456	457	432	481	483						
12. LSC t4	444	447	451	451	426	.425	429	436	448	451	447					
13. Age	490	489	487	452	468	465	463	437	495	494	484	452				
14. Gender	490	489	487	452	468	465	463	437	495	494	484	452	500			
15. MB	488	487	485	451	467	464	462	436	493	492	482	451	498	498		
16. NI	490	489	487	452	468	465	463	437	495	494	484	452	500	500	498	

*ELS* = Early Literacy Skills. *HLE* = Home Literacy Environment. *LSC* = Literacy Self-Concept. *MB* = Migration Background. NI = Nonverbal Intelligence. The numbers indicate the sample sizes used for the correlation between the respective variables. Only complete observations were used

**Table 7:** Full results of the cross-sectional multiple linear regressions with the interaction term between early literacy skills and home literacy environment on literacy self-concept

	β	*SE*	*t*	*p*
t1				
2003ELS t1	-.01	.08	-0.15	.879
HLE t1	.13	.11	1.22	.222
ELS t1*HLE t1	.06	.12	0.53	.594
Gender	.07	.05	1.45	.148
Age	.15	.05	3.15	.002
MB	.10	.05	2.09	.038
NI	-.15	.05	-3.07	.002
t2				
ELS t2	.04	.08	0.53	.598
HLE t2	.11	.11	1.03	.305
ELS t2*HLE t2	.35	.13	2.75	.006
Gender	.04	.05	0.92	.358
Age	.05	.05	1.11	.266
MB	.05	.05	1.06	.290
NI	-.07	.05	-1.49	.138
t3				
ELS t3	.09	.08	1.25	.213
HLE t3	.21	.10	2.06	.040
ELS t3*HLE t3	.25	.12	1.98	.048
Gender	-.03	.05	-0.65	.517
Age	-.03	.05	-0.56	.578
MB	.06	.05	1.22	.222
NI	-.09	.05	-1.75	.081
t4				
ELS t4	.27	.07	3.66	<.001
HLE t4	.17	.11	1.60	.110
ELS t4*HLE t4	.31	.12	2.63	.009
Gender	.01	.05	0.11	.913
Age	.19	.05	3.93	<.001
MB	.12	.05	2.46	.014
NI	.05	.05	1.14	.257

*ELS* = Early Literacy Skills. *HLE* = Home Literacy Environment. *ELS*HLE* = interaction term between ELS and HLE. *MB* = migration background. *NI* = nonverbal intelligence. ELS, HLE, and LSC were standardized prior to analyses. Only complete observations were used

**Table 8:** Correlations of all variables of interest with imputed data (*N* = 500)

	1	2	3	4	5	6	7	8	9	10	11	12	13	14	15	16
1. ELS t1																
2. ELS t2	.83^***^															
3. ELS t3	.79^***^	.85^***^														
4. ELS t4	.66^***^	.73^***^	.73^***^													
5. HLE t1	.44^***^	.41^***^	.43^***^	.28^***^												
6. HLE t2	.37^***^	.39^***^	.41^***^	.29^***^	.74^***^											
7. HLE t3	.27^***^	.29^***^	.30^***^	.19^***^	.68^***^	.70^***^										
8. HLE t4	.23^***^	.28^***^	.30^***^	.30^***^	.55^***^	.61^***^	.63^***^									
9. LSC t1	-.06	-.10^*^	-.11^*^	-.09^*^	-.06	-.01	-.03	-.01								
10. LSC t2	-.04	-.02	-.05	-.02	-.02	.01	.02	-.01	.39^***^							
11. LSC t3	.03	.04	.06	.05	-.00	.06	.08	.04	.31^***^	.38^***^						
12. LSC t4	.10^*^	.16^***^	.16^***^	.27^***^	-.00	.12^*^	-.03	.12^*^	.25^***^	.26^***^	.37^***^					
13. Age	.13^**^	.15^**^	.07	.24^***^	-.17^***^	-.11^*^	-.16^***^	-.09^*^	.12^*^	.03	-.02	.23^***^				
14. Gender	.10^*^	.08	.07	.01	.04	.12^*^	.02	.05	.05	.04	-.01	.03	-.05			
15. MB	-.37^***^	-.37^***^	-.36^***^	-.29^***^	-.27^***^	-.25^***^	-.20^***^	-.24^***^	.13^**^	.08	.02	.02	.04	-.02		
16. NI	.22^***^	.28^***^	.25^***^	.28^***^	.13^*^	.13^*^	.11^*^	.14^**^	-.14^**^	-.09^*^	-.07	.11^**^	.15^***^	.13^**^	-.06	

*ELS* = Early Literacy Skills. *HLE* = Home Literacy Environment. *LSC* = Literacy Self-Concept. *MB* = Migration Background. *NI* = Nonverbal Intelligence

^*^ *p* <.05. ^**^ *p* <.01. ^***^ *p* <.001

**Table 9:** Full results of the cross-sectional multiple linear regressions with the interaction term between early literacy skills and home literacy environment on literacy self-concept using imputed data (*N* = 500)

	β	*SE*	*t*	*p*
t1				
Intercept	-.02	.05	-0.48	.630
ELS t1	-.04	.08	-0.49	.625
HLE t1	.10	.11	0.91	.364
ELS t1*HLE t1	.14	.11	1.26	.208
Gender	.08	.04	1.89	.062
Age	.15	.05	3.28	.001
MB	.11	.05	2.26	.024
NI	-.16	.05	-3.59	<.001
t2				
Intercept	-.03	.05	-0.62	.535
ELS t2	.01	.07	0.20	844
HLE t2	.15	.11	1.40	.163
ELS t2*HLE t2	.21	.12	1.77	.077
Gender	.05	.05	1018	.238
Age	.04	.05	0.91	.364
MB	.08	.15	1.69	.092
NI	-.10	.05	-2.19	.029
t3				
Intercept	-.02	.05	-0.33	.744
ELS t3	.13	.07	1.80	.073
HLE t3	.18	.10	1.79	.074
ELS t3*HLE t3	.15	.12	1.20	.231
Gender	-.00	.05	-0.08	.933
Age	-.01	.05	0.29	.771
MB	.06	.05	1.18	.238
NI	-.10	.05	-2.06	.040
t4				
Intercept	-.03	.04	-0.62	.535
ELS t4	.38	.08	4.98	<.001
HLE t4	.25	.11	2.34	.020
ELS t4*HLE t4	.32	.14	2.25	.025
Gender	.04	.04	0.95	.345
Age	.17	.04	3.71	<.001
MB	.10	.04	2.26	.024
NI	.02	.04	0.40	.690

*ELS* = Early Literacy Skills. *HLE* = Home Literacy Environment. *ELS*HLE* = interaction term between ELS and HLE. *MB* = migration background. *NI* = nonverbal intelligence. ELS, HLE, and LSC were standardized prior to analyses

**Table 10:** Correlations of all variables of interest for children with and without a migration background

	1	2	3	4	5	6	7	8	9	10	11	12	13	14	15
1. ELS t1		.83^***^	.78^***^	.75^***^	.42^***^	.39^***^	.44^***^	.32^***^	-.13	-.12	-.09	.00	.13	.15^*^	.24***
2. ELS t2	.80^***^		.85^***^	.82^***^	.38^***^	.41^***^	.46^***^	.37^***^	-.19^**^	-.12	-.09	.06	.09	.11	.34***
3. ELS t3	.75^***^	.80^***^		.82^***^	.27^***^	.26^*^	.31^***^	.26^***^	-.22^**^	-.16^*^	-.01	.04	.04	.06	.30***
4. ELS t4	.68^***^	.73^***^	.76^***^		.28^***^	.33^***^	.36^***^	.28^***^	-.19^**^	-.14	-.05	.17^*^	.24^***^	.08	.35***
5. HLE t1	.30^***^	.25^***^	.22^***^	.18^**^		.79^***^	.79^***^	.71^***^	-.01	.05	.03	-.01	-.33^***^	.03	.08
6. HLE t2	.26^***^	.26^***^	.24^***^	.18^**^	.71^***^		.76^***^	.73^***^	-.01	-.00	.03	.14	-.17^*^	.10	.16*
7. HLE t3	.19^**^	.18^**^	.19^**^	.07	.63^***^	.65^***^		.69^***^	-.01	.00	.06	-.00	-.21^**^	.05	.05
8. HLE t4	.09	.07	.05	.05	.61^***^	.67^***^	.72^***^		.02	.01	.01	.07	-.22^**^	.04	.12
9. LSC t1	.10	.04	.06	.05	.03	.03	.03	.03		.37^***^	.25^***^	.26^***^	.14^*^	.04	-.22**
10. LSC t2	.15^*^	.12^*^	.12	.13^*^	.08	.03	.06	.03	.40^***^		.37^***^	.29^***^	.03	.07	-.14*
11. LSC t3	.20^***^	.16^**^	.17^**^	.20^**^	.12	.03	.11	.03	.42^***^	.40^***^		.35^***^	-.03	-.08	-.08
12. LSC t4	.24^***^	.22^***^	.27^***^	.32^***^	.12^*^	.09	.01	.04	.27^***^	.30^***^	.38^***^		.25^***^	-.01	.14
13. Age	.17^**^	.23^***^	.14^*^	.32^***^	-.06	-.05	-.11	-.02	.09	.04	-.01	.26^***^		-.06	.13
14. Gender	.06	.06	.03	-.07	.16^**^	.12^*^	-.00	.02	.05	.01	.01	.01	-.04		.13
15. NI	.17^**^	.21^***^	.18^**^	.21^***^	.14^*^	.13^*^	.09	.07	-.07	-.01	-.03	.13^*^	.18^**^	.11	

Lower-left coefficients represented children with migration background, upper-right coefficients represent children without migration background. *ELS* = Early Literacy Skills. *HLE* = Home Literacy Environment. *LSC* = Literacy Self-Concept. *NI* = Nonverbal Intelligence

^*^ *p* <.05. ^**^ *p* <.01. ^***^ *p* <.001

**Table 11:** Results of the cross-sectional multiple linear regressions with the interaction term between early literacy skills and home literacy environment on literacy self-concept for children with and without a migration background

	β	*SE*	*t*	*p*
t1				
Intercept	-.15/.05	.06/.09	-2.49/0.63	.013/.530
ELS t1	.17/-.24	.11/.13	1.62/-1.83	.206/.069
HLE t1	.03/.34	.16/.19	0.19/1.84	.853/.068
ELS t1*HLE t1	-.07/.09	.25/.18	-0.29/0.49	.770/.627
Gender	.04/.13	.06/.08	0.87/1.59	.383/.114
Age	.08/.27	.06/.08	1.42/3.20	.158/.002
NI	-.09/-.21	.06/.08	-1.50/-2.62	.136/.010
t2				
Intercept	-.14/-.02	.06/.09	-2.13/-0.27	.034/.785
ELS t2	.16/-.09	.11/.13	1.42/-0.67	.156/.504
HLE t2	-.05/.33	.15/.18	-0.36/1.79	.720/.076
ELS t2*HLE t2	.38/.43	.24/.19	1.56/2.21	.898/.029
Gender	.01/.11	.06/.08	0.13/1.36	.972/.175
Age	-.00/.13	.06/.08	-0.04/1.59	.653/.113
NI	.38/-.13	.06/.09	-0.45/-1.50	.120/.136
t3				
Intercept	-.11/.03	.06/.09	-1.81/0.32	.072/.747
ELS t3	.26/-.06	.10/.12	2.59/-0.56	.010/.579
HLE t3	.14/.27	.14/.19	0.99/1.44	.322/.152
ELS t3*HLE t3	.21/.20	.23/.20	0.92/0.98	.360/.327
Gender	.01/-.10	.06/.08	0.21/-1.21	.838/.227
Age	-.03/-.04	.06/.09	-0.48/-0.44	.634/.659
NI	-.06/-.10	.06/.20	-1.09/-1.16	.275/.246
t4				
Intercept	-.13/.11	-.06/.08	-2.19/1.31	.029/.192
ELS t4	.39/.14	.10/.12	3.87/1.14	<.001/.255
HLE t4	.04/.30	.15/.17	0.28/2.33	.780/.021
ELS t4*HLE t4	.18/.42	.24/.17	0.78/2.45	.349/.016
Gender	.03/-.01	.06/.08	0.48/-0.18	.635/.859
Age	.17/.24	.06/.08	2.80/2.92	.005/.004
NI	.04/.09	.06/.08	0.62/1.18	.538/.238

Values reported as *children with/without migration background. ELS* = Early Literacy Skills. *HLE* = Home Literacy Environment. *ELS*HLE* = interaction term between ELS and HLE. *NI* = nonverbal intelligence. ELS, HLE, and LSC were standardized prior to analyses. Only complete observations were used

**Table 12:** Analysis of differences in cross-sectional multiple linear regressions to predict literacy self-concept between children with and without a migration background

	β	*SE*	*t*	*p*
t1				
ELS t1	.16	.11	1.44	.150
HLE t1	.05	.16	0.33	.738
MB	.19	.10	1.80	.073
ELS t1*HLE t1	-.10	.26	-0.37	.713
ELS t1*MB	-.36	.16	-2.27	.024
HLE t1*MB	.20	.23	0.86	.388
ELS t1*HLE t1*MB	.19	.31	0.61	.541
Gender	.16	.09	1.69	.092
Age	.03	.01	3.19	.002
NI	-.01	.00	-3.00	.003
t2				
ELS t2	.15	.11	1.30	.194
HLE t2	-.04	.15	-0.23	.818
MB	.10	.11	0.91	.361
ELS t2*HLE t2	.33	.25	1.32	.189
ELS t2*MB	-.23	.16	-1.44	.151
HLE t2*MB	.33	.23	1.45	.149
ELS t2*HLE t2*MB	.09	.31	0.28	.777
Gender	.09	.09	1.00	.320
Age	.01	.01	1.08	.279
NI	-.01	.00	-1.32	.164
t3				
ELS t3	.27	.11	2.52	.012
HLE t3	.14	.14	0.92	.358
MB	.12	.10	1.22	.225
ELS t3*HLE t3	.21	.25	0.87	.387
ELS t3*MB	-.34	.15	-2.33	.020
HLE t3*MB	15	.22	0.65	.515
ELS t3*HLE t3*MB	.01	.31	0.02	.984
Gender	-.06	.09	-0.70	.487
Age	-.01	.01	-0.66	.510
NI	-.01	.00	-1.63	.103
t4				
ELS t4	.36	.10	3.63	<.001
HLE t4	.04	.15	0.28	.784
MB	.23	.10	2.33	.020
ELS t4*HLE t4	.18	.24	0.75	.456
ELS t4*MB	-.19	.14	-1.34	.180
HLE t4*MB	.34	.22	1.51	.131
ELS t4*HLE t4*MB	.24	.29	0.80	.422
Gender	.04	.09	0.23	.817
Age	.02	.01	4.03	<.001
NI	.01	.00	1.23	.202

*ELS* = Early Literacy Skills. *HLE* = Home Literacy Environment. *NI* = nonverbal intelligence. * denotes interaction between the variables. ELS, HLE, and LSC were standardized prior to analyses. Only complete observations were used
